# Feature Selection for Physical Activity Prediction Using Ecological Momentary Assessments to Personalize Intervention Timing: Longitudinal Observational Study

**DOI:** 10.2196/57255

**Published:** 2025-01-24

**Authors:** Devender Kumar, David Haag, Jens Blechert, Josef Niebauer, Jan David Smeddinck

**Affiliations:** 1 Ludwig Boltzmann Institute for Digital Health and Prevention, Salzburg, Austria; 2 Department of Psychology, Paris Lodron University of Salzburg, Salzburg, Austria; 3 Digital Health Information Systems, Center for Health & Bioresources, AIT Austrian Institute of Technology GmbH, Graz, Austria; 4 Centre for Cognitive Neuroscience, Paris Lodron University of Salzburg, Salzburg, Austria; 5 University Institute of Sports Medicine, Prevention and Rehabilitation, Paracelsus Medical University, Salzburg, Austria

**Keywords:** digital health, behavior change, tailoring, personalization, adaptive systems, ecological momentary assessments, sensing, questionnaires, machine learning, feature selection, situated research, physical activity, implementation intentions, barriers, intention-behavior gap, artificial intelligence, AI, well-being, user assessment, survey, self-efficacy, stress, mood, emotions, mobile phone

## Abstract

**Background:**

There has been a surge in the development of apps that aim to improve health, physical activity (PA), and well-being through behavior change. These apps often focus on creating a long-term and sustainable impact on the user. Just-in-time adaptive interventions (JITAIs) that are based on passive sensing of the user’s current context (eg, via smartphones and wearables) have been devised to enhance the effectiveness of these apps and foster PA. JITAIs aim to provide personalized support and interventions such as encouraging messages in a context-aware manner. However, the limited range of passive sensing capabilities often make it challenging to determine the timing and context for delivering well-accepted and effective interventions. Ecological momentary assessment (EMA) can provide personal context by directly capturing user assessments (eg, moods and emotions). Thus, EMA might be a useful complement to passive sensing in determining when JITAIs are triggered. However, extensive EMA schedules need to be scrutinized, as they can increase user burden.

**Objective:**

The aim of the study was to use machine learning to balance the feature set size of EMA questions with the prediction accuracy regarding of enacting PA.

**Methods:**

A total of 43 healthy participants (aged 19‐67 years) completed 4 EMA surveys daily over 3 weeks. These surveys prospectively assessed various states, including both motivational and volitional variables related to PA preparation (eg, intrinsic motivation, self-efficacy, and perceived barriers) alongside stress and mood or emotions. PA enactment was assessed retrospectively via EMA and served as the outcome variable.

**Results:**

The best-performing machine learning models predicted PA engagement with a mean area under the curve score of 0.87 (SD 0.02) in 5-fold cross-validation and 0.87 on the test set. Particularly strong predictors included self-efficacy, stress, planning, and perceived barriers, indicating that a small set of EMA predictors can yield accurate PA prediction for these participants.

**Conclusions:**

A small set of EMA-based features like self-efficacy, stress, planning, and perceived barriers can be enough to predict PA reasonably well and can thus be used to meaningfully tailor JITAIs such as sending well-timed and context-aware support messages.

## Introduction

In light of financial, staffing, and other pressures on societal and health care systems linked to aging populations and the increased prevalence of chronic diseases related to sedentary lifestyles and other behavioral patterns [1], a growing number of digital health apps aim to promote positive lifestyle changes [2]. Such apps have the potential to improve health outcomes [3], prevent diseases [4], and enhance the quality of life for individuals [5]. For instance, encouraging physical activity (PA) and heart-healthy habits can help prevent serious health issues like coronary heart disease, diabetes, and cancer [6]. Yet, long-term adherence to PA presents a significant challenge [7]. Despite the well-documented benefits of regular exercise for overall health and disease prevention, many people struggle to maintain consistent PA [7].

To improve the effectiveness and adherence to PA, just-in-time adaptive interventions (JITAIs) are being investigated to tailor personalized and contextualized digital health support, often enabled by mobile health technologies such as wearable sensing devices and smartphones [8,9]. JITAIs offer a promising solution to tackle issues around physical inactivity, enabling effective behavior change and habitualization by providing personalized and timely support to individuals (eg, sending motivational messages to incentivize movement after prolonged periods of inactivity). JITAIs are configured to use real-time data and context awareness to deliver the “right” interventions precisely when they are needed the most [8,9]. JITAIs based on passive sensing have already been applied to various areas including eating disorders [10], mental health conditions, obesity and weight management, PA promotion [11], and smoking cessation [12].

Tailoring JITAIs based on passively sensed contextual factors (PSCFs), such as location, activity type or levels, daily weather conditions, or an individual’s heart rate over time, is commonly observed in the literature. However, these passively sensed features face challenges in capturing relevant signals, which enable accurate and reliable characterizations of user contexts. These accurately represent what matters to a user at a given time, for example, informing the decision rules within a JITAI. To establish relevance, accuracy, and reliability, it would be necessary to validate passively sensed feature sets against the subjective user experience, difficult to do in the context of emotions and self-regulation that play a central part in the enactment of health behaviors such as PA [13]. Therefore, tailoring PA-fostering JITAIs based on PSCF comes with a considerable risk of misaligning intervention timing and content with users’ current states. Misaligned interventions may annoy users or could hinder engagement leading to drop out from JITAI use [9,14]. In literature, self-report measures of momentary affect and motivation have been shown to be closely related to actual PA [15,16]. This raises the question whether these self-reports—referred to as ecological momentary assessment (EMA)—could directly be used as an addition or alternative to tailoring JITAIs via PSCF.

The EMA can present a valuable option for collecting near-real-time information on the experiences, interests, abilities, needs, behaviors, or other contextual circumstances of individuals in their natural context [17,18]. The advantages of EMA lie in providing rich, context-specific data with reduced memory biases, facilitating a deeper understanding of human behaviors and experiences directly tied to the individual. EMAs are therefore widely used across various fields to gain insights into psychological and behavioral dynamics [18]. Although EMA has shown tremendous potential in capturing individuals’ momentary experiences, its use in PA adherence prediction has not been widely explored. Additionally, EMA comes with challenges such as participant burden and reactance [18]. Participant burden refers to the issue that asking too many questions or asking questions too frequently can leave participants annoyed, potentially causing them to ignore EMA prompts or discontinue their participation entirely [19]. Reactance, on the other hand, can be another outcome of overly frequent or extensive inquiries, which might also unduly influence participants’ perceptions or behaviors [18]. Compared to EMA deployment in limited-duration study settings, these concerns are even more relevant when EMA is intended to inform JITAI tailoring over prolonged periods of time (ie, months or years in real-world applications). Thus, alongside investigating whether EMA can be used to accurately predict PA engagement, it is critical to identify the most predictive EMA questions that allow for a sufficiently accurate prediction of PA engagement without overburdening the user.

Accordingly, we investigated whether EMA can be used to predict PA engagement and thereby inform the contextualized tailoring of JITAIs. Further, from a practical perspective, we focused on understanding design implications and strategies to balance concerns of EMA fatigue [20,21] with traditional optimization and feature selection techniques in machine learning (ML). The ML techniques can more efficiently uncover hidden patterns and relationships in the data compared to traditional survey validation methods. This work is novel in exploring the viability of EMA for the timewise tailoring of JITAIs—in terms of “when a JITAI should optimally be delivered”—and in preparing such tailoring not directly based on observed variable thresholds but on prediction outcomes. These overarching aims are reflected in the following guiding research questions (RQs):

- RQ1: How accurately can EMAs predict PA engagement or adherence?
- RQ2: Which motivational, emotional, or volitional psychological states captured through EMAs will best predict PA? What is the smallest set of predictors that balances acceptable user burden with practically sufficient prediction accuracy?

Based on the existing EMA literature [22], we expect that EMA can inform behavioral predictions as they closely reflect a participant’s perceived state, which can often be related to subsequent behavior. Regarding the constructs that could inform the prediction of PA from EMA, health behavior models, such as the Integrated Behavior Change Model [23], the Health Action Process Approach [24], or the Temporal Self-Regulation Theory [13] alongside our prior work on predictors of PA [15], suggest motivational and volitional qualities of self-regulation are closely related to PA engagement. This includes constructs such as intrinsic motivation, intention, action planning, and self-efficacy alongside anticipated contextual barriers and momentary affect (eg, current mood or stress). Therefore, we expect these factors to be strong predictors of PA engagement in this study.

## Methods

### Study Design

The EMA data used in this paper were collected in our prior investigation [15], aimed at understanding the determinants and barriers of PA engagement. Following informed consent and after completing psychometric and demographic (see Table S1 in Multimedia Appendix 1) questionnaires in an web-based survey, participants began the study’s EMA phase. During this phase, 4 EMA prompts were sent to the participants on a daily basis at fixed time points (9 AM, 1 PM, 5 PM, and 9 PM) over the course of a 3-week study period. Additionally, participants were also able to report PA independently of those fixed EMA prompts. After the 3 weeks of EMA, another web-based questionnaire was sent to the participants, to assess compliance and reactivity. However, the analyses in this study focus on the EMA data.

### Ethical Considerations

The study received ethics approval from the University of Salzburg, Austria (GZ 11/2020). Informed consent was provided by all participants prior to study enrollment and covered all primary and secondary analyses. Study participants were compensated with US $32 and received personalized feedback based on their data after the study concluded [25]. All data in this paper were anonymized.

### EMA Measures

While selecting EMA items, we aimed to align with the structure proposed in contemporary models of health behavior, such as the Health Action Process Approach [26], the Integrated Behavior Change Model for PA [23], or the Temporal Self-Regulation Theory [13]. These models propose a 2-step process to explain the implementation of health behaviors such as PA. This process consists of (1) a motivational phase, which leads to the formation of intentions and (2) a volitional phase that bridges the gap between intention and health behavior enactment.

To reflect these 2 steps in our study design, we first sampled candidate predictors proposed by these models as relevant to the motivational phase at each EMA prompt. This included measures of momentary mood (10 items from the Positive and Negative Affect Schedule [27]: happy, relaxed, active, irritated, concerned, depressed, nervous, stressed, energetic, and tired) and stress :2 items from the Perceived Stress Scale (PSS) [28]. The German version by Schneider et al [29] anticipated barriers to PA (“How well would your given circumstances allow you to be physically active at the moment?”; BarrPA), and pain as a barrier to PA (“At the moment, do you have physical complaints that impede physical activity?”). Furthermore, the morning prompt contained items assessing sleep quality (“How good was your sleep?”; SleepQlt), the time of falling asleep (“When did you fall asleep?”; ST*)*, and the waking time (“When did you wake up in the morning?”; WT), which could also be either barriers or resources for PA engagement.

Next, we prospectively asked the participants about their intentions to be physically active (“Do you intend to be physically active in the next 4 hours?,” yes or no). Only if they responded with “yes,” we further assessed volitional determinants of health behavior enactment such as planning specificity (“How specifically did you plan this physical activity?”; ActPlan), self-efficacy (“How strongly do you believe you can enact your plan under the given circumstances?”; ActPlanSE), and momentary intrinsic motivation (“Independent of the circumstances, how motivated are you right now to be physically active?”). Given the practical purpose around supporting adherence or enactment of planned and intended PA, selecting for positive PA intention provides reasonable grounds.

All of these items were developed or adjusted to fit the specific needs of this study. Except intention and sleep duration, they were measured on a horizontal slider from 0-not at all to 100-very much. Within each EMA prompt, participants finally reported their PA retrospectively, (“Have you been physically active in the last 4 hours?,” yes or no), which we used as the primary outcome in our analyses (see Haag et al [15] for cross-validation of these PA self-reports against wearable data). For this EMA sampling, we first used the Smarteater app (internally developed by the University of Salzburg), and in a later recruitment phase, switched to the m-Path platform [30]. However, each participant only used 1 platform, and assessments were identical on both platforms. Therefore, data quality was not impaired due to switching between the two platforms. For the full list of EMA items, please refer to Table S2 in Multimedia Appendix 1.

### Study Participants

Participants were healthy individuals who self-reported having no limitations in their ability to perform PA, and did not describe themselves as competitive athletes. As of June 2022, a total of 49 participants were enrolled in the data collection, which was conducted in a phased manner. Of these, 10 participants dropped out before completing all 3 weeks. However, the data from 4 of the 10 dropouts were still used for this investigation since they filled out enough EMA prompts to be included over 1 week. The final sample included 43 participants (31 female and 12 male) between 19 -67 years of age (mean 39.14, SD 15.53) years. In total, 16 reported high, 22 moderate, and 5 low activity levels in the International Physical Activity Questionnaire [31] completed at the beginning of the study. Based on the participants’ self-reported height and weight, their BMI ranged from 19.0 to 39.6 (mean 24.4, SD 3.4) kg/m^2^, with 2 participants being obese (BMI≥30.0 kg/m^2^), 11 overweight (BMI=25.0‐29.9 kg/m^2^), and 29 with normal weight (BMI=18.5‐24.9 kg/m^2^). The BMI for 1 participant was omitted due to being unrealistic (ie, unreasonable self-reported height and weight).

For further details on study setup, procedure, as well as the primary outcomes, please see Haag et al [15].

### Data Cleaning and Feature Engineering

Each EMA prompt contained at least 16 questions. Some questions in the EMA prompts included conditional subquestions (eg, planning specificity only when a PA was intended) Questions requiring per-day-level measurements (eg, sleep quality and duration) were limited to 1 morning prompt per day. This approach resulted in 41 distinct questions that were assessed in the EMA. For practical reasons, we only considered the prospective and momentary questions as predictors of retrospectively assessed PA in the subsequent analysis since they are better suited for modeling the likelihood of upcoming PA adherence at runtime. Further details of the EMA questions can be found in Table S2 in Multimedia Appendix 1.

During the data cleaning process, EMA questions with less than a 30% response compliance rate were dropped as data augmentation on such low compliance rates could introduce a bias in the models [32]. EMA prompts for which participants did not report whether they performed the PA were also discarded; this was done retrospectively. For example, if a participant did not report engaging in PA within the last 4 hours at 1 PM, their 9 AM prompt data were excluded from the analysis. The times participants fell asleep and woke up were self-reported once only in the first EMA of the day. Therefore, empty cells for sleep and wake time for EMA prompts 2, 3, and 4 were populated with values from the first EMA of each day. Any remaining missing values for sleep hours, wakeup time, and sleep qualitywhen participants did not respond to the day’s first EMA prompt were replaced with their respective median values up to that point in time. The categorical EMA questions with “yes or no” answers were transformed to binary values (1/0). The StandardScaler from the *Scikit-learn* package [33] was used to scale all the continuous variables to unit variance. This standardization of input features can be beneficial for the performance and convergence of ML algorithms.

### Model Training and Validation

For modeling, a representative selection of common ML methods, including logistic regression, decision tree, support vector machine [34], k-nearest neighbor (KNN) [35], random forest (RF) [36], and extreme gradient boosting (XGBoost or XGB) [37] was used to compare the model prediction performances. The decision to choose traditional ML models for exploration over neural networks was made, given the relatively small size of the dataset, since neural networks typically require an extensive dataset for training. The data were split into training or test sets (in a ratio of 80:20) using the *Scikit-learn* package [33]. A stratified 5-fold cross-validation method on the training dataset was used to evaluate model performance and hyperparameter tuning [38]. The stratified k-fold cross validation method was chosen to ensure that each fold’s class distribution similar to the overall class distribution in the dataset. This is particularly useful when dealing with imbalanced datasets, where one class may have significantly more instances than the others. All models were then evaluated on the test dataset. The GridSearchCV scoring method from *Scikit-learn* [33] was used to find the optimal hyperparameters.

Model prediction performances were compared using the area under the receiver operating characteristic curve, as it is a more appropriate measure compared to simple accuracy, especially for imbalanced datasets [39]. The area under the receiver operating characteristic curve visualizes the performance of the model at various probability thresholds for classification and helps in selecting an appropriate threshold that balances the trade-off between true positives and false positives.

To overcome data imbalance, we attempted upsampling with the synthetic minority oversampling technique (SMOTE) [40] that is implemented in the Python library *imbalanced-learn* [41] on the training data. We trained the models with the upsampled data and compared their internal validation results on the test dataset.

For identifying the top features predicting whether PA took place within a given EAM slot, we used the recursive feature elimination (RFE) technique from *Scikit-learn* [33]. RFE is primarily used in ML to select the most relevant and important features from a given dataset. It recursively trains the model using subsets of features and ranks them based on their contribution to improving prediction outcomes. By iteratively eliminating less important features, RFE provides an optimal subset of features that maximizes the model’s performance. After the RFE, we used the Shapley Additive Explanations (SHAP) framework [42] for understanding the feature importance within the best-performing models. The models were built in Python 3.8 using *Scikit-learn* (version 1.2.0) package [33]. The entire training process was conducted on a Windows 11 operating system with 64 GB RAM, a dual-core Intel Core i9 processor, and an Nvidia graphics card (Nvidia RTX A4000) with 16 GB memory.

## Results

### Overview

Following data cleaning, 23 of 41 EMA questions fulfilled the minimum data compliance requirements and were used as features for building the models and predicting the compliance or noncompliance of PA. In this section, we first outline the performance of the range of selected candidate ML models in predicting PA engagement when all the features were used in training. Thereafter, we will show the results of feature importance in predicting PA engagement using the RFE technique and a SHAP value visualization. We conclude with a practical discussion on selecting an appropriate model or ensemble and feature set, contextualizing the work around more general concerns in model training and selection for dynamic personalization based on EMA.

### PA Prediction Performance of Various Model Using All Available Features

**Figure 1.**
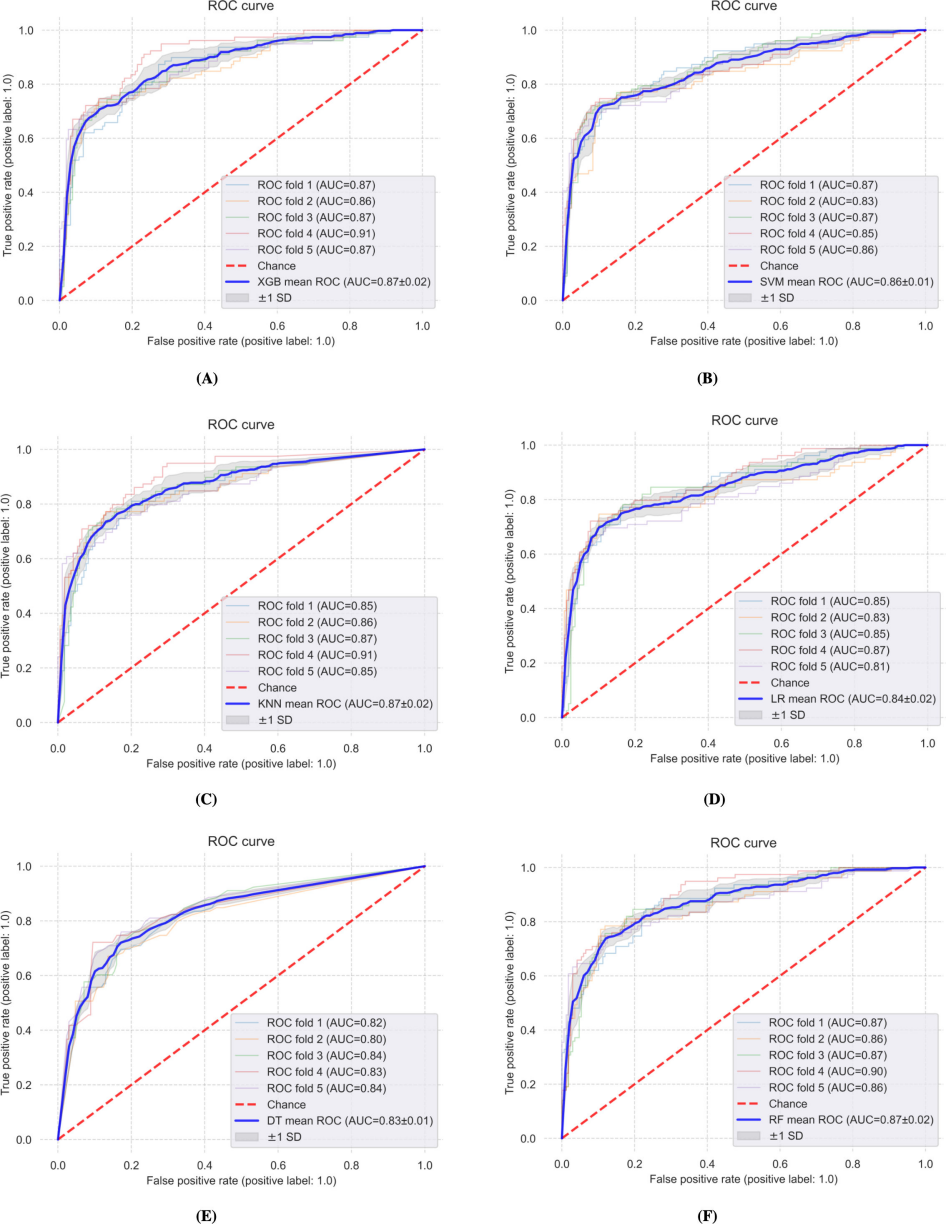
(**A**) XGB, (**B**) SVM, (**C**) KNN, (**D**) LR, (**E**) DT, and (**F**) RF show the performance of selected machine learning models in 5-fold cross-validation using all the features. AUC: area under the curve; DT: decision tree; KNN: k-nearest neighbor; LR: logistic regression; RF: random forest; ROC: receiver operating characteristic curve; SVM: support vector machine; XGB: extreme gradient boosting.

Figure 1 shows the area under the receiver operating characteristic curve of various models in a 5-fold cross-validation on training data, and Table 1 presents their area under the curve (AUC) scores on training and test sets. XGB, KNN, and RF models have shown the best performance and achieved a mean AUC score of 0.87 (SD 0.02) on 5-fold cross-validation. However, among them, XGB achieves a slightly higher AUC score (0.87) on the unseen test set. We also tested the upsampling technique SMOTE on the training data; however, as shown in the 2 rightmost columns in Table 1, this did not significantly improve the test set AUC score. Since SMOTE does not improve the performance on the test set, it was not used for further analysis.

**Table 1.** Performance of various models on 5-fold cross-validation and test set^a^.

Model name	AUC^b^ score 5-fold cross-validation, mean (SD)	AUC score on test set	SMOTE AUC score 5-fold cross-validation, mean (SD)	SMOTE AUC score on test set
XGB^c^	0.87 (0.02)	0.87	0.93 (0.01)	0.86
RF^d^	0.87 (0.02)	0.86	0.94 (0.01)	0.86
KNN^e^	0.87 (0.03)	0.86	0.92 (0.01)	0.85
LR^f^	0.84 (0.02)	0.85	0.86 (0.02)	0.83
SVM^g^	0.86 (0.02)	0.86	0.86 (0.03)	0.84
DT^h^	0.83 (0.01)	0.83	0.86 (0.01)	0.81

a Contrasted with performance following synthetic minority oversampling technique (SMOTE) [8] of training data.

b AUC: area under the curve.

c XGB: extreme gradient boosting.

d RF: random forest.

e KNN: k-nearest neighbor.

f LR: logistic regression.

g SVM: support vector machine.

h DT: decision tree.

### Feature Importance

#### Overview

To reduce potential questionnaire fatigue [20] caused by the extensive EMA survey length and finding the smallest but accurate subset of EMA questions for predicting the PA, after finalizing the best-performing model on all 23 features, we focused on understanding the feature importance in predicting PA. Since the XGB model showed slightly better performance on the test dataset (Table 1) compared to RF and KNN, explorations into feature importance were done exclusively for XGB .

#### Recursive Feature Elimination

**Figure 2.**
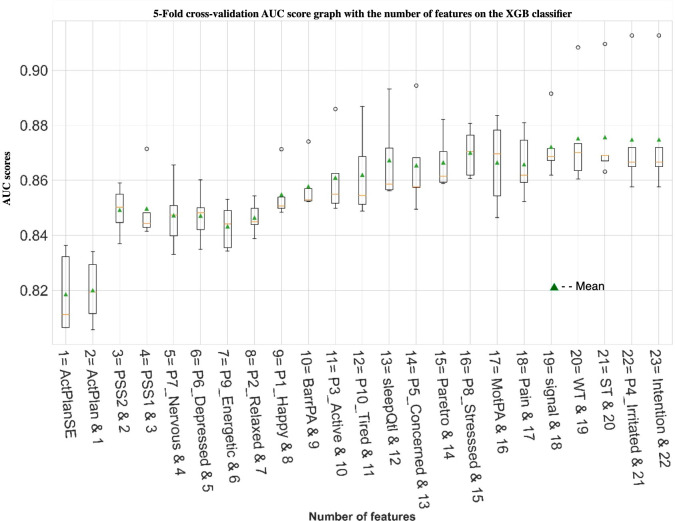
XGB models with growing feature set sizes: boxplots of AUC scores with a number of features using recursive feature elimination. The numbers (1, 2, 3, etc) indicate the top n-number of features and their names. Feature sets are listed as “additonal_feature & X (identifier of compounded prior features).” AUC: area under the curve; XGB: extreme gradient boosting.

Figure 2 shows the selected top-n (n=number 1, 2, 3, etc) features identified by RFE, alongside with a box plot of the corresponding AUC scores for the XGB classifier. As depicted in Figure 2 , for the XGB models, the highest mean AUC score (0.87) is achieved when using 13 features and remains the same even when all 23 features are used. Interestingly, a competitive AUC score of 0.85 is achieved with just 3 features, which combine ActPlan and ActPlanSE as indicators of volitional intention, along with PSS2 as a marker of stress. An increase to an AUC score of 0.86 is only achieved when using 10 or more features.

#### SHAP Value–Based Feature Importance

**Figure 3.**
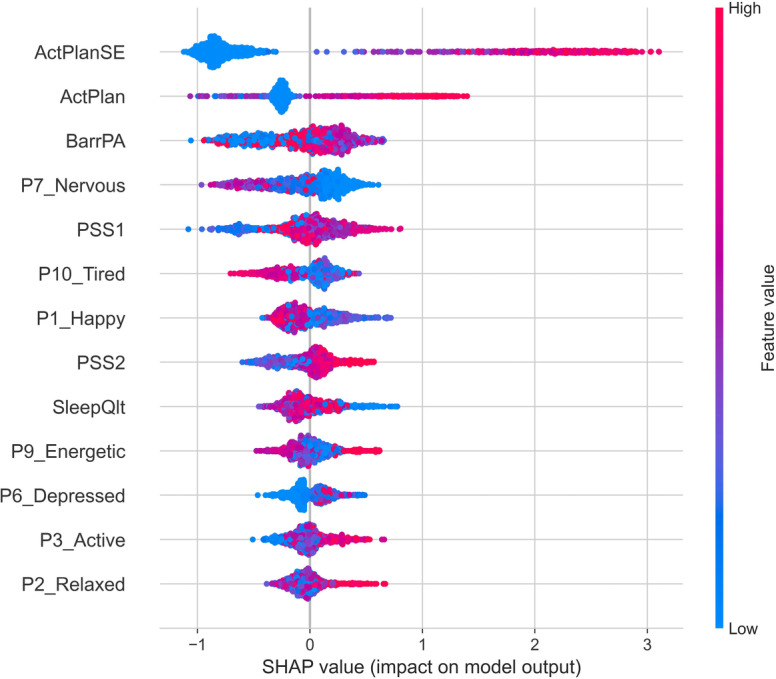
SHAP values graph of top 13 features indicating how each feature is contributing to the extreme gradient boosting model’s output. SHAP: Shapley Additive Explanations.

Figure 3 shows a SHAP value graph of the top 13 features and their contributions in PA prediction by the XGB model. For brevity, we present SHAP values of 13 features, as subsequent features do not improve the AUC score (as apparent in Figure 2). SHAP value graph indicates the correlations through which different features contribute to the model’s output for individual predictions.

Similar to the RFE (Figure 2), items ActPlanSE (self-efficacy) and ActPlan (PA planning specificity) remain the top 2 features with the most notable impact on model output (indicated by wide impact score spread combined with decisive directionality on the horizontal axis). The positive SHAP values indicate that the feature increases the PA enactment prediction, while negative values indicate the opposite. The color of the bar represents the feature value (red for high and blue for low). The slight difference in the feature ranking by RFE and SHAP value is due to differences in their feature selection methodology and objective of feature selection. RFE focuses on improving overall model performance metrics, whereas SHAP values aim to provide interpretable explanations for individual prediction by a given feature.

## Discussion

### Principal Findings

In this study, we explored the viability of predicting PA engagement based on common supervised ML methods with features drawn from EMA data collected in the participants’ natural contexts. Conceptually, if PA engagement or nonengagement can be predicted, this can inform the timing of issuing JITAIs. For instance, if negative engagement is predicted, motivational messages of encouragement, or more specific planning or replanning, can be sent. Since EMA requires the active involvement of the user, we also explored which features or questions of the EMA were key determinants for effectively predicting PA engagement. This exploration offers a framework for informing trade-off decisions that can help in designing JITAI systems that take valuable information derived from EMA without overburdening the users.

### EMA and PA Engagement Prediction

Regarding RQ1 on how accurately EMAs and ML models can help predict PA engagement, all the ML models (Table 1) produce AUC scores of 80 or higher on test data that were not seen by the models in training. When considering smaller performance differences, both XGB and RF models performed best on both cross-validation (mean 0.87, SD 0.2) and test sets (AUC 0.87 and 0.86, respectively), broadly indicating that the questions used in the current EMA could be a viable option for PA engagement prediction and can complement the tailoring of passive sensing–based JITAIs to foster PA.

While such performance levels would not satisfy strong reliability requirements (eg, in health diagnostics), they can arguably be reasonably used in JITAI settings around fostering long-term PA, where occasional type 1 or type 2 errors do not have grave consequences. Compared to previous work on the same dataset, which used non-ML models to investigate determinants of PA enactment in a theory-driven manner [15], this study adopts a practical approach on the potential of operationalizing EMA for actively driving JITAIs independently of preconditions, such as the PA being explicitly intended. Thereby, favoring model performance over analyses intended to inform theory building for feature composition.

The outcomes of automated feature selection corroborate earlier findings [15] with non-ML modeling, as feature importance analysis indicates that self-efficacy (ActPlanSE) and planning specificity (ActPlan) are the key relevant predictors of PA engagement. Here, self-efficacy represents a participant’s confidence in their ability to engage in the intended activity, and planning specificity refers to how specifically they had planned that activity. This result also falls in line with other previous publications indicating a close relationship between PA engagement and these volitional components [43-45]. Further, analysis based on RFE on the XGB model (Figure 2) indicates stress (PSS1 and PSS2) as another potentially relevant factor that can be a qualifier for PA outcomes. This association of stress and PA is also consistent with previous EMA literature [16].

### Psychological Considerations and Contextualization in Theory

In light of the dual-process models of health behavior [13,23] that the present data collection was based upon, it is not surprising to see the volitional constructs (ActPlanSE and ActPlan) ending up having greater feature importance. However, even though we found a resemblance to these psychological models, our results are not to be confused with an evaluation of such models. Instead, our findings represent a practical investigation of the ML methodology being applied to determine which ones (within a given set of candidate EMA items) could be used for a runtime system to predict PA adherence and control adaptive interventions accordingly. This implies that the proposed feature selection might be very different in another set of potential predictors and necessitates the interpretation of our findings in light of our specific EMA design.

For example, consider the intention item or feature that was ranked at the very bottom of our RFE (Figure 2). From a theoretical point of view, intention would often be seen as the pivotal point in health behavior engagement [24]. Therefore, it may seem very surprising that intention (Figure 2) is being ranked the least predictive feature in our dataset. This discrepancy originates in the structure of our EMA, with ActPlanSE and ActPlan only being assessed if participants report to have the intention for PA. Therefore, these features would have missing values for each episode where no intention is reported. In these cases, values of the volitional determinants were replaced with zeros. Thus, the binary intention data are encoded in ActPlanSE and ActPlan. In the RFE process, the presented models will have picked up on this characteristic and ranked intention itself very low since it does not contain additional information if ActPlan or ActPlanSE is included. However, this does not imply that we can forgo the assessment of intention since it is only meaningfully possible to assess planning and self-efficacy when an intention is given. In practical terms, this would imply that the precondition of intention being given would be wrapped into the wording for an ActPlan item if it were to be presented without an explicit item on intention preceding it (ie, “In case you are intending to exercise, how specifically have you planned”).

Nonetheless, the indicated feature combination including ActPlan and stress items corresponds well with expectations that can be derived from the aforementioned dual-process models of health behavior [13,23,26], representing elements that can be seen to capture aspects of both a motivational phase and a volitional phase.

### Trade-Off Between EMA Burden and PA Prediction Accuracy

The potential viability of the sets of EMA questionnaires selected for this study was informed by psychological theories. However, the list of questions as a whole is clearly too burdensome for being of practical use in guiding JITAIs. As indicated in the EMA literature [19-21], questionnaire fatigue or overload is a real concern for practicality. As shown in Figure 2, an AUC of over 0.85 is achieved by just using the top 3 features (ie, ActPlanSE, ActPlan, and PSS2) as compared to the maximal achievable AUC of 0.87 when all the 23 features were used. Moreover, as evidenced by the RFE process, the maximal AUC score of 0.87 is already achieved with 13 features, indicating that beyond these 13 features, the rest other features are not adding additional benefit for predicting PA engagement. Even within these 13 features, after adding an item from the stress questions (PSS1 and PSS2), Positive and Negative Affect Schedule mood questions (P7_nerves, P5_concern, P1_happy, P6_depressed, and P7_energetic) are arguably not adding significant improvements in AUC score to justify their inclusion as frequently asked items in a practical longer-term deployment. The PA-friendly external circumstances (BarrPA) and the absence of barriers and sleep quality (SleepQlt) do minor improvement of the AUC score and could be candidates for inclusion with the EMA for research purposes but would likely not be included in a production system, as the improvements are negligible for practical purpose.

For most practical purposes and keeping the EMA questionnaire burden to the minimum, in settings where an intention for PA is given (eg, supporting the engagement of a PA plan), a deployed JITAI model could be driven by just 3 EMA items (ActPlanSE, ActPlan, and PSS2). If the intention is not given, the first item could be rephrased as indicated earlier.

### Design Implications

For the practicality of JITAIs or any intervention intended for fostering PA to be driven by regular EMA in live deployment situations, it is essential to select EMA questions effectively and avoid causing EMA fatigue. In relation to RQ2, about which concepts will best inform PA engagement prediction (as shown in the Feature Importance section), features (EMA questions) related to self-efficacy, planning, and stress have reasonable PA prediction power. These outcomes indicate that the long list of potentially relevant EMA questions used in PA prediction can be effectively cut short to a degree where real-world deployments of JITAIs with decision rules or trigger points [14] being informed by EMAs appear plausible. In addition, the proposed XGB model can be integrated into existing digital health platforms or mobile apps for effective EMA-based JITAIs and fostering PA engagement.

### Limitations and Future Work

This study is formative with regard to surveying potentially relevant EMA constructs and measures. It does not include observations or testing of the app of the derived models in practical deployment, which is a clearly indicated step for future work. The study also included a relatively small number of participants (n=43) and a limited time frame (3 weeks). To ensure the generalizability of the ML model (XGB) for automated physical activity prediction and the customization of JITAIs across diverse demographics, further research with larger sample sizes and longer study durations is needed. Additionally, in this study setup, the EMA frequency was established at 4 times a day. Future work should explore the impact of varying EMA frequencies per day on the predictive capabilities of the model for PA.

### Conclusions

This paper presents a formative investigation into EMA’s effectiveness in predicting PA implementation and whether EMA can be of practical use in gathering temporal context for JITAI decision-making. The outcomes with common supervised ML models, especially XGB with a mean AUC score of 0.87 (SD 0.02), indicate that EMA can offer relevant PA prediction power and thereby has the potential to complement more common passive sensing JITAI-tailoring approaches. Furthermore, the investigation around finding the right trade-off between EMA question load and PA engagement, prediction-accuracy of ML models indicates that self-efficacy (ActPlanSE) and planning specificity (ActPlan) play the most important role in determining the PA prediction under the assumption that initial intention to perform PA was already given. Three features, ActPlanSE, ActPlan, and PSS2 (stress), allowed for achieving an AUC score of 0.85 as compared to the maximum AUC score of 0.87 when all the 23 EMA questions were used.

## Supplementary material

10.2196/57255 Multimedia Appendix 1 A Detailed list of ecological momentary assessment (EMA) questions used in the study and list of questionnaires included in the pre- and post-EMA surveys.

## Abbreviations

AUC: area under the curve
EMA: ecological momentary assessment
JITAI: just-in-time adaptive intervention
KNN: k-nearest neighbor
ML: machine learning
PA: physical activity
PSCF: passively sensed contextual factor
PSS: Perceived Stress Scale
RF: random forest
RFE: recursive feature elimination
RQ: research question
SHAP: Shapley Additive Explanations
SMOTE: synthetic minority oversampling technique
XGB: extreme gradient boosting
