# Supplementary material for: Feature Selection for Physical Activity Prediction Using Ecological Momentary Assessments to Personalize Intervention Timing: Longitudinal Observational Study
Source: JMIR Mhealth Uhealth. 2025 Jan 24;13:e57255. doi: 10.2196/57255 (PMC11785349; doi:10.2196/57255)
Supplement: Multimedia Appendix 1 [file mhealth-v13-e57255-s001.docx]

## **Table S1.** List of questionnaires included in the pre- and post-EMA surveys.

| Pre-EMA-Survey | Post-EMA-Survey |  |
| --- | --- | --- |
| Demographics | Compliance |  |
| Subjective Health Status | Reactivity |  |
| Health Satisfaction |  |  |
| International Physical Activity Questionnaire (IPAQ) [1] |  |  |
| Intention for Change [2] |  |  |
| Assessment of HAPA Stadium [2] |  |  |
| Preactional Self-Efficacy [2] |  |  |
| Maintenance Self-Efficacy [2] |  |  |
| Recovery Self-Efficacy [2] |  |  |
| Outcome Expectancies [2] |  |  |
| Risk Perception [2] |  |  |
| Salzburg Stress Physical Activity Scale (SSPAS; adapted from Salzburg Stress Eating Scale [3]) |  |  |
| Prospective Planning [2] |  |  |
| Behavioural Regulation in Exercise Questionnaire (BREQ-3) [4], [5] |  |  |

## **Table S2.** List of all EMA items used in the present study.

| EMA Items | | | |
| --- | --- | --- | --- |
| Variable | Abbreviation | Item | Scale |
| Moring Prompt | | | |
| Sleep Quality | sleepQlt | How good was your sleep? | 0 (very bad) – 100 (very good) |
| Time of falling asleep | ST | When did you fall asleep? | Time |
| Waking time | WT | When did you wake up in the morning? | Time |
| All Prompts | | | |
| Stress (PSS; [6]) | PSS1 | Do you feel like you have everything under control? | 0 (not at all) – 100 (very much) |
|  | PSS2 | Do you feel like you can handle all upcoming tasks and challenges appropriately? | 0 (not at all) – 100 (very much) |
| Mood (PANAS; [7]) | How do you feel right now? | | |
|  | P1_Happy | Happy | 0 (not at all) – 100 (very much) |
|  | P2_Relaxed | Relaxed | 0 (not at all) – 100 (very much) |
|  | P3_Active | Active | 0 (not at all) – 100 (very much) |
|  | P4_Irritated | Irritated | 0 (not at all) – 100 (very much) |
|  | P5_Concerned | Concerned | 0 (not at all) – 100 (very much) |
|  | P6_Depressed | Depressed | 0 (not at all) – 100 (very much) |
|  | P7_Nervous | Nervous | 0 (not at all) – 100 (very much) |
|  | P8_Stressed | Stressed Out | 0 (not at all) – 100 (very much) |
|  | P9_Energetic | Energetic | 0 (not at all) – 100 (very much) |
|  | P10_Tired | Tired | 0 (not at all) – 100 (very much) |
| Barriers | Pain | At the moment, do you have physical complaints that impede physical activity? | 0 (not at all) – 100 (very much) |
|  | BarrPA | How well would your given circumstances allow you to be physically active at the moment? | 0 (not at all) – 100 (very much) |
| Intention | Intention | Do you intend to be physically active in the next four hours? | Yes or No |
| ‘Intention_yes’ | | | |
| Planning | ActPlan | How specifically did you plan this physical activity? | 0 (not at all) – 100 (very much) |
| Self-Efficacy | ActPlanSE | How strongly do you believe, you can enact your plan under the given circumstances? | 0 (not at all) – 100 (very much) |
| Intrinsic Motivation | MotPA | Independent of the circumstances, how motivated are you right now to be physically active? | 0 (not at all) – 100 (very much) |
| All Prompts | | | |
| Retrospective PA | | Have you been physically active in the last four hours? | Yes or No |
| ‘PA_yes’ | | | |
| Activity Type | | What was your physical activity? | Selection (Cycling, Running, Workout, Walking, Hiking) or Free Text Input |
| Activity Duration | | When did you start your activity? | Time |
|  |  | When did you finish your activity? | Time |
| Intense Minutes | | Overall: How many minutes have you been active with high exertion? (e.g. heavily breathing, strongly increased pulse) | Minutes |
| Moderate Minutes | | Overall: How many minutes have you been active with moderate exertion? (e.g. breathing a little harder, slightly increased pulse) | Minutes |
| Satisfaction | | How satisfied were you with this activity? | 0 (not at all) – 100 (very much) |
| Enjoyment | | How much did you enjoy this activity? | 0 (not at all) – 100 (very much) |
| Support | | What helped you to be active? | Free Text Input |
| Company | | I was active … | Alone or With Others |
| Evening Prompt | | | |
| Influences | | How much influence did the following areas have on you being physically active today? | |
|  |  | Family | -100 (prevented) – 0 (not influenced) – 100 (supported) |
|  |  | Work | -100 (prevented) – 0 (not influenced) – 100 (supported) |
|  |  | Social Contacts | -100 (prevented) – 0 (not influenced) – 100 (supported) |
|  |  | Physical Condition | -100 (prevented) – 0 (not influenced) – 100 (supported) |
|  |  | Mood | -100 (prevented) – 0 (not influenced) – 100 (supported) |
|  |  | Appointments / Leisure Time | -100 (prevented) – 0 (not influenced) – 100 (supported) |
| Intention (Day-Level) | | Do you plan to be physically active tomorrow? | Yes or No |
| ‘Day-Level Intention_yes’ | | | |
| Planning (Day-Level) | | Have you planned this activity specifically (time, place, circumstances) or are you allowing it to happen? | 0 (Spontaneously) – 100 (Very Precisely Planned) |
| Intense Minutes Planned | | How many minutes do you plan to be active with high exertion? (e.g. catching your breath, highly increased pulse) | Minutes |
| Moderate Minutes Planned | | How many minutes do you plan to be active with moderate exertion? (e.g. breathing a little harder, slightly increased pulse) | Minutes |

[1] C. L. Craig *u. a.*, „International physical activity questionnaire: 12-country reliability and validity“, *Med. Sci. Sports Exerc.*, Bd. 35, Nr. 8, S. 1381–1395, Aug. 2003, doi: 10.1249/01.MSS.0000078924.61453.FB.

[2] R. Schwarzer, „HAPA - Assessment Tools“. Zugegriffen: 8. Februar 2024. [Online]. Verfügbar unter: http://userpage.fu-berlin.de/%7Ehealth/hapa_assessment.pdf

[3] A. Meule, J. Reichenberger, und J. Blechert, „Development and preliminary validation of the Salzburg Stress Eating Scale“, *Appetite*, Bd. 120, S. 442–448, Jan. 2018, doi: 10.1016/j.appet.2017.10.003.

[4] P. M. Wilson, W. M. Rodgers, C. C. Loitz, und G. Scime, „“It’s Who I Am … Really!’ The Importance of Integrated Regulation in Exercise Contexts1“, *J. Appl. Biobehav. Res.*, Bd. 11, Nr. 2, S. 79–104, Apr. 2006, doi: 10.1111/j.1751-9861.2006.tb00021.x.

[5] D. Markland und V. Tobin, „A Modification to the Behavioural Regulation in Exercise Questionnaire to Include an Assessment of Amotivation“, *J. Sport Exerc. Psychol.*, Bd. 26, Nr. 2, S. 191–196, Juni 2004, doi: 10.1123/jsep.26.2.191.

[6] E. E. Schneider, S. Schönfelder, M. Domke-Wolf, und M. Wessa, „Measuring stress in clinical and nonclinical subjects using a German adaptation of the Perceived Stress Scale“, *Int. J. Clin. Health Psychol.*, Bd. 20, Nr. 2, S. 173–181, Mai 2020, doi: 10.1016/j.ijchp.2020.03.004.

[7] D. Watson, L. A. Clark, und A. Tellegen, „Development and Validation of Brief Measures of Positive and Negative Affect: The PANAS Scales“, S. 8, doi: 10.1037/0022-3514.54.6.1063.
